# Obesity Does Not Interfere with the Cholesterol-Lowering Effect of Plant Stanol Ester Consumption (as Part of a Heart-Healthy Diet)

**DOI:** 10.3390/jcdd8040036

**Published:** 2021-04-07

**Authors:** Piia Simonen, Elisa Arte, Helena Gylling

**Affiliations:** 1 Heart and Lung Center, Cardiology, Helsinki University Hospital and University of Helsinki, P.O. Box 350, 00029 HUS Helsinki, Finland; piia.simonen@hus.fi; 2 Raisio Nutrition Ltd., P.O. Box 101, 21201 Raisio, Finland; elisa.arte@raisio.com

**Keywords:** atherosclerosis, cholesterol, cholesterol absorption, cholesterol synthesis, coronary artery disease, LDL-cholesterol, obesity, overweight, plant stanol ester, sitostanol

## Abstract

Dietary modifications including plant stanol ester consumption are recommended measures to control serum and low-density lipoprotein (LDL)-cholesterol concentrations, but obesity can affect their responses. We investigated whether body mass index (BMI) affects serum cholesterol levels during plant stanol (mainly sitostanol) ester consumption. This ad hoc analysis was based on earlier results of a cross-over, randomized controlled trial of postmenopausal women consuming rapeseed oil-based margarine without or with plant stanol ester (3 g plant stanols/day) for seven weeks. We classified the subjects as normal-weight (BMI ≤ 25 kg/m^2^, *n* = 9, mean 22.6 kg/m^2^) or overweight/obese (BMI > 25 kg/m^2^, *n* = 11, mean 28.4 kg/m^2^), and recalculated the results, focusing on cholesterol absorption, cholesterol synthesis, and fecal steroid outputs. Serum cholesterol levels were similar in the groups during the control diet. Plant stanol ester reduced serum cholesterol by 0.63 ± 0.19 mmol/L (11%) in normal-weight and by 0.75 ± 0.13 mmol/L (12%) in overweight/obese subjects (*p* < 0.05 for both), and cholesterol absorption was reduced in both groups. However, relative and dietary cholesterol absorption were more effectively reduced in normal-weight subjects. In conclusion, overweight/obesity did not interfere with the serum cholesterol response to plant stanol ester consumption despite substantial differences in cholesterol metabolism between the groups.

## 1. Introduction

Cardiovascular diseases (CVDs) cause more disability-adjusted life-years (DALYs) and deaths than any other disease group globally [1,2]. In 2017, CVDs caused almost 18 million deaths, of which about 12 million were caused by atherosclerotic cardiovascular diseases (ASCVDs), i.e., coronary-artery disease (CAD), ischemic stroke, and peripheral-artery disease [1]. Thus, ASCVD is still an important risk factor as regards human health. The etiology of ASCVD is different from that of the other CVDs, because low-density lipoproteins (LDLs) cause ASCVD [3,4].

Recent studies also highlighted the surprising frequency of subclinical atherosclerosis in apparently healthy middle-aged subjects with a low risk of ASCVD [5,6,7,8,9,10]. Subclinical atherosclerosis can proceed without symptoms to clinically evident ASCVD in a few years in connection with circulating concentrations of low-density lipoprotein cholesterol (LDL-C) [5,6,7,8,9,10]. However, its progression, as well as the risk of ASCVD events, can be prevented by lowering LDL-C concentrations [9,11,12,13,14]. 

Dietary modifications including plant stanol ester consumption are recommended measures to control LDL-C at a population level, and in hypercholesterolemic subjects at risk of ASCVD, the latter in combination with drug treatment [15]. Plant stanols, when consumed at a rate of two to three grams a day as fatty-acid esters, diminish the absorption of cholesterol by about 41–44% [16] and lower LDL-C concentrations on average by 0.33–0.42 mmol/L (9–12%) [17]. The cholesterol-lowering mechanism of plant stanols and plant sterols differs from those of other cholesterol-lowering dietary components by providing additive cholesterol lowering when combined with other dietary components. For example, plant stanol esters used as part of a heart-healthy diet can lower LDL-C concentrations on average by 1.45 mmol/L (35%) compared with the habitual diet [18]. Plant stanols reduce the absorption of both cholesterol and plant sterols and can thus preferentially be used in subjects with the gene variants in ABCG5/G8 transporters that induce both elevated cholesterol and plant sterol absorption, leading to doubling of the risk of CAD [19]. The overall incidence of these gene variants is relatively high, about 30% at a population level, so the dietary use of plant stanols provides a safe way of mitigating the implied risk. 

Obesity can affect the serum cholesterol and LDL-C responses in dietary interventions [20]. Thus, the substitution of unsaturated for saturated fatty acid intake reduced serum cholesterol, LDL-C, and apoprotein B (apo B) concentrations more effectively in normal-weight than in obese subjects [21]. The question arises of whether or not plant stanol esters lower serum cholesterol concentrations less in obese than in normal-weight subjects, since obesity markedly modifies cholesterol and LDL-apo B metabolism [22,23,24,25,26].

This study is an ad hoc analysis of an original randomized, controlled cross-over trial, in which the effects of plant stanol ester consumption were evaluated in connection with serum lipids, lipoproteins, and cholesterol and lipoprotein metabolism in postmenopausal women with CAD [16]. For the present study, the original study population was classified into normal-weight and overweight/obese subjects, and the results concerning serum cholesterol and variables of cholesterol metabolism during control and intervention periods were calculated in connection with the two groups. The primary outcome measures were the obesity-related differences in serum cholesterol and in variables of cholesterol metabolism at the end of control and plant stanol ester diet periods. The secondary outcome measures were the relative changes brought about by use of plant stanol ester consumption as compared to those of the placebo period in the respective variables in both groups.

## 2. Materials and Methods

### 2.1. Study Population

The original study involved 22 successive postmenopausal women with angiographically verified CAD treated at Helsinki University Central Hospital in 1994 [16]. The subjects were studied in a steady state at least three months after myocardial infarction or a coronary-artery procedure. The subjects had no liver, thyroid, or renal problems, or diabetes mellitus, and no lipid-lowering treatment or estrogen-replacement therapy. In 1994, drug-based lipid-lowering therapy was not a routine regimen for CAD patients in Finland. The mean age of the subjects was 51 years (range 48–56 years), their mean weight 68 kg (range 53–86 kg), and mean body mass index (BMI) 26 kg/m^2^ (range 21–33 kg/m^2^. 

The present study population consisted of 20 subjects, because the original data for two subjects were not available for recalculation. The subjects were classified into normal-weight (BMI ≤ 25 kg/m^2^) and overweight/obese (BMI > 25 kg/m^2^) groups. 

### 2.2. Study Procedure

**Figure 1:**
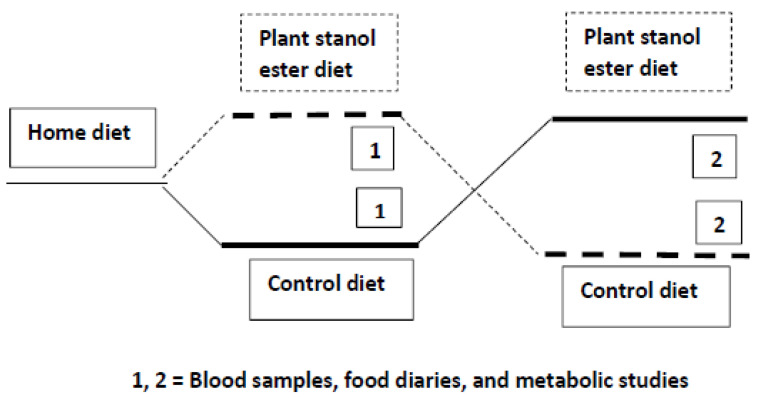
The study procedure. Each period lasted for 7 weeks.

A detailed description of this randomized, placebo-controlled, double blind cross-over trial is available in Ref. [16]. A condensed scheme of the present study comparing the end results between the active and control periods in the two BMI groups is presented in Figure 1.

In brief, after a run-in period on their regular home diets, the subjects replaced in a randomized order 21 g of their daily total fat intake with a rapeseed oil-based margarine without (control diet) or with plant stanol ester (3 g plant stanols/day)(plant stanol ester diet) for seven weeks, followed by switching of the margarines for another 7-week period without a wash-out period. Since an earlier plant stanol ester intervention has demonstrated that a steady state is achieved within 1–2 weeks without any carry-over effect [27], a wash-out period was considered unnecessary in this 7 + 7- week intervention. The plant stanols were mainly comprised of sitostanol, the most frequent plant stanols in food. The subjects were advised to use margarine daily in three divided doses during each of the three major meals. 

Blood samples were drawn after a 12-h fast at the end of the 7-week diet periods (Figure 1). At the same time the metabolic studies were performed with a focus on cholesterol absorption [28], whole-body cholesterol synthesis, and fecal-steroid outputs. For this purpose, the subjects were given capsules to take three times a day for the last seven days of the periods, each capsule containing ^14^C-cholesterol, ^3^H-sitosterol, and Cr_2_O_3_. During these periods, each subject also kept a 7-day food diary. Three-day fecal collections were carried out at the end of the 7-day treatment periods. 

All subjects gave written informed consent. The study was performed according to the principles of the Declaration of Helsinki. The Ethics Committee of the Second Department of Medicine, University of Helsinki approved the study protocol both to the basic and follow-up study. 

### 2.3. Methods

Quantitation of serum concentrations of cholesterol, lathosterol, and sitosterol was performed using gas–liquid chromatography (GLC) with a 50-m capillary column (Ultra 2, Agilent Technologies, Wilmington, DE, USA) and flame ionization detection with 5α-cholestane as internal standard [29]. The samples from different times per subject were analyzed in the same GLC run. Serum concentrations of lathosterol and sitosterol were adjusted to those of cholesterol in the same GLC run and expressed as ratios to cholesterol (10^2^ μmol/mmol cholesterol) to enable comparison between subjects with different LDL levels. Serum lathosterol and sitosterol ratios to cholesterol are validated biomarkers of cholesterol synthesis and cholesterol absorption efficiency [30,31,32,33]. Cholesterol synthesis was analyzed by using the sterol-balance technique. Fecal neutral sterols, bile acids, and plant stanols were analyzed by GLC [34]. Fecal neutral sterols denote excretion of cholesterol and cholesterol-derived compounds to the feces, and fecal bile acids depict their de novo synthesis. Dietary intakes of cholesterol and fatty acids were analyzed by way of a computerized method, using data from the 7-day food diaries [35].

### 2.4. Calculations 

Cholesterol synthesis = (fecal elimination of cholesterol as neutral sterols plus bile acids) − dietary cholesterol intake.

Total intestinal cholesterol pool = fecal neutral sterols/(100 − cholesterol absorption efficiency).

Dietary intestinal cholesterol pool = dietary cholesterol intake.

Biliary intestinal cholesterol pool = total intestinal cholesterol pool − dietary cholesterol intake.

Total cholesterol absorbed = cholesterol absorption efficiency × total intestinal cholesterol pool/100.

Dietary cholesterol absorbed = cholesterol absorption efficiency × dietary cholesterol intake.

Biliary cholesterol absorbed = Total cholesterol absorbed − Dietary cholesterol absorbed.

### 2.5. Statistics

Statistical analyses were performed by using SPSS for Windows 22.0 (SPSS, Chicago, IL, USA). Sample-size calculation was based on significance levels (a = 0.05 and b = 0.20) and essential information obtained from the previous study [16]. Using these estimates, the size of the required population was 21, suggesting that the size of the present study population was appropriate. Normality and homogeneity of variance assumptions were checked before further analyses, and variables not normally distributed were transformed logarithmically. Continuous variables were tested by using Student’s *t*-test. Variables not normally distributed even after logarithmic transformation, or nonhomogeneous in variance, were tested by using Mann–Whitney U-tests. A two-sided *p*-value of <0.05 was considered statistically significant. The results are expressed as means ± SEs.

## 3. Results

### 3.1. Serum Sterols and Variables of Cholesterol Metabolism during the Control Diet

Of the twenty subjects, nine belonged to the normal-weight (BMI ≤ 25 kg/m^2^) and 11 to the overweight/obese (BMI > 25 kg/m^2^) group (Table 1). The age of the subjects did not differ between the groups. The dietary intakes of cholesterol and total fatty acids were similar in the two groups. Fecal plant stanol concentrations, robustly depicting their dietary intake because of their low absorption of <0.2% [36], were also similar in the two groups.

Serum cholesterol concentrations were mildly to moderately elevated and similar in the groups (Table 2). Serum triglyceride levels were within the normal range in both groups (0.9 ± 0.2 mmol/L, BMI ≤ 25 kg/m^2^ and 1.5 ± 0.3 mmol/L, BMI > 25 kg/m^2^). Measures of cholesterol absorption efficiency, serum sitosterol, and dietary cholesterol absorbed were lower in the overweight/obese subjects compared with the normal-weight subjects. The amounts of biliary and total cholesterol absorbed were not affected by BMI. 

Measures of cholesterol synthesis, serum lathosterol, fecal neutral sterols denoting the excretion of cholesterol and cholesterol-derived compounds to the feces, and biliary and total intestinal cholesterol pools were higher in the overweight/obese subjects than in the normal-weight subjects (Table 2). The dietary cholesterol pool and fecal bile-acid concentrations were identical in the two groups.

### 3.2. Plant Stanol Ester Consumption, Serum Sterols, and Variables of Cholesterol Metabolism

Plant stanol ester margarine was well tolerated and no side effects were reported. Dietary intakes of cholesterol and total fatty acids were similar in the normal-weight and overweight/obese subjects, as they were in the control period (Table 1). Fecal plant stanol concentrations increased from the control diet values to about 2600 mg/day during the plant stanol ester diet (Table 1). The amounts of fecal plant stanols ranged between 1729–3529 mg/day in the normal-weight subjects and 1836–3421 mg/day in the overweight/obese subjects.

**Figure 2:**
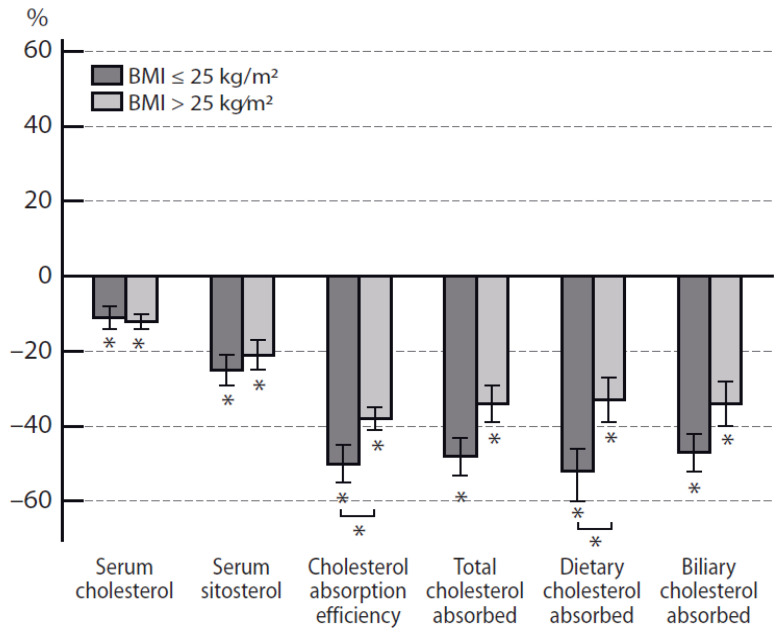
Relative changes (mean ± SE) caused by the plant stanol ester diet compared with the control diet in serum cholesterol concentrations and variables of cholesterol absorption in the study population divided into normal-weight (BMI ≤ 25 kg/m^2^) and overweight/obese (BMI > 25 kg/m^2^) subjects. * *p* < 0.05 between the plant stanol ester vs. the control diet, └*┘ *p* < 0.05 between the BMI groups.

Serum cholesterol levels were reduced similarly in both groups, by 0.63 ± 0.19 mmol/L (11%) in the normal-weight subjects and by 0.75 ± 0.13 mmol/L (12%) in the overweight/obese subjects (*p* < 0.05 for both) (Table 2, Figure 2). Serum triglyceride concentrations were unchanged from the control-diet values and similar in the two groups (1.0 ± 0.2 mmol/L, BMI ≤ 25 kg/m^2^ and 1.4 ± 0.2 mmol/L, BMI > 25 kg/m^2^). 

Measures of cholesterol absorption efficiency, serum sitosterol, and dietary, biliary, and total cholesterol absorbed decreased after intake of dietary plant stanols in both groups (Table 2). However, relative cholesterol absorption efficiency and dietary cholesterol absorbed decreased more in the normal-weight than in the overweight/obese subjects (Table 2, Figure 2).

Measures of cholesterol synthesis and fecal neutral sterols increased only in the normal-weight subjects, although they remained at lower levels than in the overweight/obese subjects (Table 2). Serum lathosterol concentrations showed only a tendency to reflect changes in cholesterol synthesis. Intestinal cholesterol pools remained practically unchanged in both groups. Hence the biliary and total intestinal cholesterol pools remained larger in the overweight/obese subjects than in the normal-weight subjects. Fecal bile acid levels were unchanged in both groups. 

## 4. Discussion

This randomized, cross-over. and placebo-controlled intervention shows that an overweight/obese condition did not affect serum cholesterol lowering by means of plant stanol ester consumption. In addition, obesity-induced elevated cholesterol synthesis did not increase serum cholesterol concentrations. 

Our novel findings demonstrated that consuming plant stanol esters by an intake of 2.6 g of plant stanols/day (Table 1; fecal plant stanols) significantly lowered serum cholesterol concentrations by 0.63 and 0.75 mmol/L (11% and 12%) in the normal-weight and overweight/obese subjects in spite of substantial differences in cholesterol metabolism between the groups. Relative and absolute cholesterol absorption diminished in both groups during the plant stanol ester diet compared with the control period. However, the relative cholesterol absorption efficiency and dietary cholesterol absorbed were more effectively reduced in the normal-weight than in the overweight/obese subjects. Cholesterol synthesis and excretion of cholesterol and cholesterol-derived compounds to the feces, denoted as fecal neutral sterols, increased significantly only in the normal-weight subjects. Nevertheless, cholesterol synthesis, fecal neutral sterols, and the total and biliary intestinal cholesterol pools remained larger during the plant stanol ester diet in the overweight/obese subjects than in the normal-weight subjects. 

During the control diet, cholesterol metabolism differed in the normal-weight and overweight/obese subjects more or less as described in earlier studies [22,23,24,25,26,37]. The only differences in the present vs. earlier studies were the lower amount of dietary cholesterol absorbed in the overweight/obese than in the normal-weight subjects and the similar levels of fecal bile acids in the two groups. Instead, plant stanol-induced effects on serum cholesterol concentration and on variables of cholesterol metabolism in normal-weight subjects were similar as described earlier [16,38,39,40,41]. Thus, it can be concluded that this study population was adequate and also large enough for statistical analyses, in conformity with the power calculation and earlier studies. However, if the research methodology would be less demanding for both the participants and for the laboratory, a larger number of especially obese subjects would have been optimal. 

Why is it then that plant stanol esters lowered serum cholesterol concentrations similarly in the two groups in spite of differences in cholesterol metabolism, and why is it that the elevated level of cholesterol synthesis in obese subjects did not result in higher blood cholesterol values? According to what we know of cholesterol homeostasis, low cholesterol absorption upregulates hepatic cholesterol synthesis. Upregulated cholesterol synthesis is in general associated with upregulated expression of LDL receptors. In fact, upregulated expression of LDL receptors leading to increased catabolism of LDL-apo B has been demonstrated in obese vs. normal-weight subjects [42]. In combination with changes in very-low-density lipoprotein (VLDL)-apo B metabolism, typical of obesity, increased LDL-apo B catabolism together with decreased LDL-apo B production in obesity result in similar serum cholesterol concentrations in obese and normal-weight subjects [42]. Since also plant stanol ester consumption increases LDL-apo B catabolism [43], these findings appeared to explain the similar serum cholesterol concentrations in the two groups in this study during both dietary periods. 

An interesting finding regarding cholesterol absorption was the surprisingly similar amounts of total absolute cholesterol absorbed daily in the normal-weight and overweight/obese subjects during both diet periods. Plant stanol ester consumption resulted in a higher daily level of absolute absorption of cholesterol (mean of 49 mg more) in the overweight/obese subjects compared with the normal-weight subjects and this was reflected in reduced fecal excretion of cholesterol and its metabolites in the overweight/obese subjects (54 mg/day less).

Effective reduction of dietary cholesterol in particular may be of importance from a cardioprotective point of view. It was recently shown that each additional intake of 300 mg of dietary cholesterol/day predicted a 17% higher risk of incident CVD and an 18% higher risk of all-cause mortality [44]. Further, in the same study, consuming half an egg/day, corresponding to 93 mg of cholesterol, on average, predicted a 6% higher risk of incident CVD and an 8% higher risk of all-cause mortality. Thus, it is possible that the amount of dietary cholesterol has a role in atherosclerosis, the mechanism of which remains to be evaluated further. 

Even though the cholesterol-lowering efficacy of plant stanol esters was comparable in the two groups with a similar plant stanol dose, the large intestinal cholesterol pool in obese or morbidly obese subjects may dilute the concentration of plant stanols in the intestine and diminish their cholesterol-lowering efficacy. In fact, in this study only a limited number of subjects were obese (BMI 30–34 kg/m^2^) and no one was morbidly obese (BMI ≥ 35 kg/m^2^). A larger dose of plant stanols may be needed in obese and morbidly obese subjects. Daily intake of plant stanols dose-dependently reduces serum concentrations of plant sterols, these representing a reliable biomarker of cholesterol absorption during plant stanol ester use [45]. Thus, since 2.0 g/day of plant stanols as esters lower LDL-C levels by 0.33 mmol/L in normal-weight subjects [17], in obesity the respective dose should be increased to 3.0 g/day to keep the intestinal plant stanol to cholesterol ratio equal to that in normal-weight subjects. This calculation was based on total intestinal pool sizes in normal-weight and obese subjects during a habitual home diet [22].

Our study had the following limitations. The number of subjects was limited although it can be considered of adequate size as regards the power calculation, and the similarity of the results to those of earlier studies dealing with cholesterol metabolism in obesity in general and plant stanol ester consumption-derived metabolic changes. However, whether or not plant stanol ester consumption also lowers serum levels of total and LDL-C in obese and morbidly obese subjects and without modifying the plant stanol dose remains to be evaluated further. It should be acknowledged that conducting a large clinical study with the same measurements of cholesterol metabolism, including the sterol balance technique, would be challenging to execute today.

In conclusion, we demonstrated here that plant stanol ester consumption effectively lowers serum cholesterol concentrations in overweight/obese postmenopausal women with CAD compared with normal-weight controls. Consumption of plant stanols as fatty acid esters as part of a heart-healthy diet is a recommended means of controlling LDL-C concentrations in order to prevent the development of subclinical atherosclerosis and ASCVD at a population level, and in subjects at risk of ASCVD in combination with drug treatment. Especially combined with statin treatment, a plant stanol or plant sterol-enriched diet significantly lowers serum cholesterol and LDL-C concentrations by 0.30 mmol/L compared with statins alone [46]. However, more information is needed in connection with morbid obesity, especially as regards adequate doses of plant stanols in these subjects. Finally, regarding the recent information that an increase in dietary cholesterol intake is associated with the risk of ASCVD events, a reduction of dietary cholesterol absorption through the use of plant stanol esters might turn out to be a valuable health benefit added to its LDL-C-lowering effect. Thus, the use of plant stanol esters is recommended in subjects with gene variants that increase cholesterol absorption, leading to an increased risk of CAD.

## Figures and Tables

**Table 1:** Characteristics of the study population divided into normal-weight (BMI ≤ 25 kg/m^2^) and overweight/obese (BMI > 25 kg/m^2^) subjects during the control and plant stanol ester diets.

Variables	Normal-Weight Subjects, *n* = 9		Overweight/Obese Subjects, *n* = 11	
	Control Diet	Plant-Stanol-ester Diet	Control Diet	Plant-Stanol-ester Diet
Age, years	53 ± 0.7		52 ± 0.8	
Weight, kg	58 ± 1	58 ± 1	74 ± 2 *	74 ± 2 *
BMI, kg/m^2^	22.6 ± 0.4	22.4 ± 0.5	28.4 ± 0.7 *	28.4 ± 0.7 *
Dietary cholesterol, mg/day	231 ± 26	220 ± 34	185 ± 20	200 ± 24
Dietary total fatty acids, g/day	73 ± 54	70 ± 6	60 ± 4	57 ± 4
Fecal plant stanols, mg/day	28 ± 13	2629 ± 296 ^†^	39 ± 21	2596 ± 239 ^†^

Mean ± SE. BMI = body mass index. * *p* < 0.05 vs. normal-weight subjects, ^†^ *p* < 0.05 vs. the control diet.

**Table 2:** Serum sterol concentrations and variables of cholesterol metabolism in the study population divided into normal-weight (BMI ≤ 25 kg/m^2^) and overweight/obese (BMI > 25 kg/m^2^) subjects at the end of the control and plant stanol ester diets.

Variables	Control Diet	Plant-Stanol-Ester Diet
Serum cholesterol, mmol/L		
BMI ≤ 25 kg/m^2^, *n* = 9	5.82 ± 0.30	5.19 ± 0.38 *
BMI > 25 kg/m^2^, *n* = 11	6.00 ± 0.28	5.24 ± 0.22 *
*p*-value ^1^	0.849	0.518
Serum lathosterol ^2^		
BMI ≤ 25 kg/m^2^, *n* = 9	139 ± 14	165 ± 19
BMI > 25 kg/m^2^, *n* = 11	189 ± 14	206 ± 20
*p*-value ^1^	0.037	0.138
Serum sitosterol ^2^		
BMI ≤ 25 kg/m^2^, *n* = 9	185 ± 19	137 ± 16 *
BMI > 25 kg/m^2^, *n* = 11	116 ± 12	89 ± 9 *
*p*-value ^1^	0.017	0.030
Cholesterol absorption efficiency, %		
BMI ≤ 25 kg/m^2^, *n* = 9	49 ± 3	24 ± 3 *
BMI > 25 kg/m^2^, *n* = 11	37 ± 2	22 ± 1 *
*p*-value ^1^	0.007	0.543
Total cholesterol absorbed ^3^		
BMI ≤ 25 kg/m^2^, *n* = 9	479 ± 46	246 ± 35 *
BMI > 25 kg/m^2^, *n* = 11	483 ± 53	295 ± 15 *
*p*-value ^1^	0.569	0.382
Dietary cholesterol absorbed ^3^		
BMI ≤ 25 kg/m^2^, *n* = 9	113 ± 13	58 ± 14 *
BMI > 25 kg/m^2^, *n* = 11	67 ± 7	43 ± 5 *
*p*-value ^1^	0.007	0.790
Biliary cholesterol absorbed ^3^		
BMI ≤ 25 kg/m^2^, *n* = 9	365 ± 39	188 ± 24 *
BMI > 25 kg/m^2^, *n* = 11	416 ± 52	252 ± 15 *
*p*-value ^1^	0.425	0.074
Total intestinal cholesterol pool ^3^		
BMI ≤ 25 kg/m^2^, *n* = 9	966 ± 65	986 ± 60
BMI > 25 kg/m^2^, *n* = 11	1322 ± 143	1333 ± 62
*p*-value ^1^	0.044	0.003
Dietary intestinal cholesterol pool ^3^		
BMI ≤ 25 kg/m^2^, *n* = 9	230 ± 26	220 ± 34
BMI > 25 kg/m^2^, *n* = 11	185 ± 20	200 ± 24
*p*-value ^1^	0.184	0.732
Biliary intestinal cholesterol pool ^3^		
BMI ≤ 25 kg/m^2^, *n* = 9	735 ± 51	766 ± 48
BMI > 25 kg/m^2^, *n* = 11	1137 ± 141	1133 ± 59
*p*-value ^1^	0.004	0.001
Fecal bile acids ^3^		
BMI ≤ 25 kg/m^2^, *n* = 9	316 ± 37	324 ± 36
BMI > 25 kg/m^2^, *n* = 11	349 ± 30	305 ± 28
*p*-value ^1^	0.425	0.518
Fecal neutral sterols ^3^		
BMI ≤ 25 kg/m^2^, *n* = 9	487 ± 40	740 ± 44 *
BMI > 25 kg/m^2^, *n* = 11	839 ± 99	1038 ± 56
*p*-value ^1^	0.002	0.002
Cholesterol synthesis ^3^		
BMI ≤ 25 kg/m^2^, *n* = 9	572 ± 54	844 ± 63 *
BMI > 25 kg/m^2^, *n* = 11	1003 ± 111	1143 ± 58
*p*-value ^1^	0.001	0.006

Mean ± SE. BMI = body mass index. Fecal neutral sterols = excretion of cholesterol and cholesterol-derived compounds to feces. ^1^ Mann–Whitney U-test between the BMI groups, ^2^ 10^2^ μmol/mmol cholesterol, ^3^ mg/day. * *p* < 0.05 vs. the control diet, paired *t*-test.

## Data Availability

The data presented in this study are available in Biomedicum Helsinki 1 A4 23b.
